# CCL20 is up-regulated in non-alcoholic fatty liver disease fibrosis and is produced by hepatic stellate cells in response to fatty acid loading

**DOI:** 10.1186/s12967-018-1490-y

**Published:** 2018-04-24

**Authors:** Xin Chu, Qunyan Jin, Hui Chen, G. Craig Wood, Anthony Petrick, William Strodel, Jon Gabrielsen, Peter Benotti, Tooraj Mirshahi, David J. Carey, Christopher D. Still, Johanna K. DiStefano, Glenn S. Gerhard

**Affiliations:** 1 Geisinger Obesity Research Institute, Geisinger Clinic, Danville, PA 17822 USA; 2 Lewis Katz School of Medicine at Temple University, Philadelphia, PA 19140 USA; 3 Translational Genomics Institute (TGEN), Phoenix, AZ USA; 4 Department of Medical Genetics and Molecular Biochemistry, Lewis Katz School of Medicine at Temple University, 960 Medical Education and Research Building (MERB), 3500 N. Broad Street, Philadelphia, PA 19140 USA

**Keywords:** NAFLD, Fibrosis, CCL20

## Abstract

**Background:**

Nonalcoholic fatty liver disease (NAFLD) is a prevalent complication of extreme obesity. Loading of the liver with fat can progress to inflammation and fibrosis including cirrhosis. The molecular factors involved in the progression from simple steatosis to fibrosis remain poorly understood.

**Methods:**

Gene expression profiling using microarray, PCR array, and RNA sequencing was performed on RNA from liver biopsy tissue from patients with extreme obesity. Patients were grouped based on histological findings including normal liver histology with no steatosis, lobular inflammation, or fibrosis, and grades 1, 2, 3, and 4 fibrosis with coexistent steatosis and lobular inflammation. Validation of expression was conducted using quantitative PCR. Serum analysis was performed using ELISA. Expression analysis of hepatocytes and hepatic stellate cells in response to lipid loading were conducted in vitro using quantitative PCR and ELISA.

**Results:**

Three orthogonal methods to profile human liver biopsy RNA each identified the chemokine CCL20 (CC chemokine ligand 20 or MIP-3 alpha) gene as one of the most up-regulated transcripts in NAFLD fibrosis relative to normal histology, validated in a replication group. CCL20 protein levels in serum measured in 224 NAFLD patients were increased in severe fibrosis (p < 0.001), with moderate correlation of hepatic transcript levels and serum levels. Expression of CCL20, but not its cognate receptor CC chemokine receptor 6, was significantly (p < 0.001) increased in response to fatty acid loading in LX-2 hepatic stellate cells, with relative increases greater than those in HepG2 hepatocyte cells.

**Conclusions:**

These results suggest that expression of CCL20, an important inflammatory mediator, is increased in NAFLD fibrosis. CCL20 serves as a chemoattractant molecule for immature dendritic cells, which have been shown to produce many of the inflammatory molecules that mediate liver fibrosis. These data also point to hepatic stellate cells as a key cell type that may respond to lipid loading of the liver.

**Electronic supplementary material:**

The online version of this article (10.1186/s12967-018-1490-y) contains supplementary material, which is available to authorized users.

## Background

Obesity is a major risk factor for the development of several metabolic disorders including nonalcoholic fatty liver disease (NAFLD), a group of conditions defined histologically that include lipid accumulation in the liver (steatosis), some degree of inflammation (steatohepatitis), and scarring (fibrosis). The overconsumption of calories leads to storage of triglycerides in adipocytes, as well as in hepatocytes in susceptible individuals. Progression of NAFLD from simple steatosis to hepatic inflammation and liver fibrosis is a complex multi-cellular process involving the interaction of a number of cell types including hepatocytes, Kupffer cells, hepatic stellate cells, sinusoidal endothelial cells as well as infiltrating immune cells [1]. The signals that regulate this complex multicellular process have not yet been well delineated [2] but likely involve a number of “hits” [3], including oxidative stress such as lipid peroxidation and mitochondrial dysfunction [4], production of pro-inflammatory mediators, such as cytokines and chemokines [5], and the intestinal microbiome producing hepatotoxic substances such as lipopolysaccharide [6, 7].

Acknowledging the molecular and cellular complexity of the pathophysiological processes that are involved in progressing from simple steatosis to more advanced forms of NAFLD [8], various “omics” approaches have been used including genomic [9, 10], transcriptomic [11], proteomic [12], lipidomic [13], and metabolomics [14, 15]. We applied several orthogonal genome-wide transcriptional profiling methods to RNA isolated from liver biopsies obtained from patients with extreme obesity undergoing bariatric surgery. We used microarray technology, a focused PCR-based array, and RNA sequencing to identify the C-C motif chemokine ligand 20 (CCL20) as a highly up-regulated transcript in NAFLD-associated fibrosis relative to normal liver histology. Circulating CCL20 protein levels in serum were found to be significantly increased in patients with liver fibrosis. To investigate a potential cellular mechanism, CCL20 gene expression was found to be markedly up-regulated in cultured human hepatic stellate cells relative to hepatocyte cells in response to fatty acid loading. CCL20 is the primary ligand for the CC chemokine receptor 6 (CCR6) and serves as a chemoattractant molecule for immature dendritic cells, which have been shown to produce many of the inflammatory molecules that mediate liver fibrosis [16]. CCL20 produced by stellate cells in response to lipid loading may therefore be a key mechanism in the fibrotic progression of NAFLD in response to the increased caloric intake in extreme obesity. These data also suggest that lipid sensing by hepatic stellate cells [17] may be a novel cellular mechanism in the progression of NAFLD. CCL20 may also be a common pathogenetic mediator in both non-alcoholic and alcoholic liver disease [18].

## Methods

### Patients and samples

Liver biopsies were obtained from Caucasian individuals enrolled in the Bariatric Surgery Program at the Geisinger Clinic Center for Nutrition and Weight Management [19]. Liver wedge biopsies were obtained intraoperatively as previously described [20–22] with a portion fixed in neutral buffered formalin, stained with hematoxylin and eosin, and histologically evaluated as part of clinical standard of care using NASH CRN criteria [23], as previously described [24]. Patients with serologic, histologic, or other evidence for any chronic liver diseases were excluded from this study. Comprehensive (1–2 h) in-person interviews and psychological evaluations were conducted by certified psychology professionals that included assessment of drug, alcohol, and smoking behaviors to determine clinically significant alcohol intake. Patients determined to have either definite or possible evidence of any addictive behavior were excluded as surgical candidates. In addition, available clinical data, including medical and medication history and diagnostic ICD-9 diagnostic codes, were searched for evidence of drug or alcohol abuse. Samples from patients with evidence of abuse were also excluded from the study. Clinical data were obtained from an obesity database as described previously [25], and included demographics, clinical measures, ICD-9 codes, medical history, medication use, and common lab results. All study participants provided written informed consent for research, which was conducted according to The Code of Ethics of the World Medical Association (Declaration of Helsinki). The Institutional Review Boards (IRBs) of Geisinger Health System and the Translational Genomics Research Institute approved the research.

### RNA isolation and cDNA synthesis

Total RNA was isolated using the RNeasy total RNA isolation kit (Qiagen, Valencia, CA,) according to the manufacturer’s protocol and quantified using the Nanodrop ND-1000 spectrophotometer (Thermo Scientific, Wilmington, DE).

### Affymetrix expression profiling

Total RNA was analyzed using the GeneChip human genome U133 plus 2.0 array (Affymetrix, Santa Clara, CA), covering > 47,000 transcripts and variants from the human genome. Due to the built-in redundancy in the microarray design, multiple probe sets mapping to the same transcript yielded a total of 39,000 interrogated loci. All reagents used to make the final hybridization cocktail were from the GeneChip Expression 3′ Amplification One-Cycle Target Labeling and Control Reagents Kit (Affymetrix). Biotin-labeled RNA fragments were produced from a pool of liver RNA with equal amounts from each of nine patients per group pooled based on liver pathology via double-stranded cDNA synthesis followed by in vitro transcription and fragmentation. Hybridization cocktails containing fragmented cRNA, array controls (Affymetrix), bovine serum albumin (BSA), and herring sperm DNA were prepared and hybridized to the array at 45 °C for 16 h. The hybridized arrays were washed, and bound biotin-labeled cRNA detected with a streptavidin–phycoerythrin conjugate. Washing and staining procedures were automated using the Affymetrix Fluidics Station (model 450). Each array was scanned once using the Affymetrix Gene Array Scanner 3000 and the Affymetrix GeneChip Operating Software v1.2 was used to adjust for artifacts, noise, and background. The global method of scaling/normalization was used to for each chip. Fluorescent signal values from the Affymetrix platform were exported to the Spotfire Decision Suite 8.1 software application (https://www.spotfire.com) for analysis. Normalization for chip-to-chip variation and probe-to-probe variability was performed. A Z-score and ANOVA were performed to identify differentially expressed genes.

### Superarray analysis

Total RNA samples were converted to cDNA with the RT^2^ First Strand Kit (SuperArray Bioscience Corp., Frederick, MD) and examined for cytokine expression level using the Human Inflammatory Cytokines and Receptors PCR Array (Cat. # PAHS-011, SuperArray Bioscience Corp., Frederick, MD) with the RT^2^ SYBR Green/ROX qPCR Master Mix (SuperArray Bioscience Corp., Frederick, MD) on a 7300 Real-Time PCR System (Applied Biosystems, Foster City, CA) according to the product protocol. Fold change was calculated by determining the ratio of mRNA levels to control values using the Δ threshold cycle (Ct) method (2^−ΔΔCt^). All data were normalized to GAPDH expression.

### Quantitative PCR (qPCR)

Total RNA derived from human tissue samples was reverse-transcribed to cDNA using QuantiTect Rev. Transcription Kit (Qiagen). Quantitative RT-PCR assays were performed in duplicate using designed TaqMan assays for human CCL20 (Hs00171125_m1), CCR6 (Hs00171121_m1), and GAPDH (Hs99999905_m1), all from Life Technologies, in conjunction with the ABI 7500 Fast Real-Time PCR system (Life Technologies, Carlsbad, CA, USA) according to the manufacturer’s protocol. Results were analyzed using the 7500 Software v2.0.6 and DataAssist v3.0 (Life Technologies).

For cell culture analysis, total RNA was extracted from cells with TRIzol reagent (ThermoFisher), and gene expression was measured using commercial TaqMan assays- CCL20 (Hs00355476_m1), CCR6 (Hs01890706_S1) and GAPDH (Hs02758991_g1) (ThermoFisher) and the TaqMan RNA-to-CT 1-step kit and the Step One Plus real–time PCR system. Gene expression levels were normalized to GAPDH and fold-change was determined using the comparative threshold method (ΔΔCT). Two or more replicate experiments were performed.

### ELISA

Serum CCL20 protein levels were measured using commercially available Human CCL20/MIP-3α Colorimetric Sandwich ELISA kits (Cat. # DM3A00, R & D Systems, Minneapolis, MN) according to manufacturer’s protocol (standard curve shown in Additional file 1: Figure S4) using triplicate measurements. Insufficient serum was available for replicate experiments.

### Cell culture

HepG2 (ATCC HB-8065) cells were culture in Eagle’s minimum essential medium (ATCC) with 10% FBS. LX-2 cells (SCC064, Millipore) were cultured in DMEM with 10% FBS. Cells were maintained at 37 °C with humidified air and CO_2_ (5%).

### Free fatty acid treatment

Sodium palmitate (2 mmol/l) (P9767, Sigma) was conjugated to fatty acid-free BSA (A7030, Sigma) in a 6:1 molar ratio as described previously [26]. For free fatty acid stimulation, cells were seeded on six-well plates and cultured in complete growth medium to what approximately 80% confluency. Cells were washed twice with pre-warmed phosphate-buffered saline, and treated with various concentrations of free fatty acid diluted into serum–free media, and incubated at 37 °C for 24 h. Experiments were performed in duplicate using triplicate measurements for each experimental condition.

### Oil red O staining

Confluent cell monolayers were fixed in phosphate-buffered paraformaldehyde (10%) for 10 min, rinsed with water, followed by 60% isopropyl alcohol, and stained with Oil Red O solution (six parts saturated Oil Red O dye in isopropyl alcohol to four parts water) for 15 min. Stained cells were washed with water, and then incubated for 5 min with 1.0 ml of isopropyl alcohol to dissolve stained oil droplets. The absorbance of the dye-triglyceride complex was measured at 500 nm.

### Statistical analysis

Determination of statistical differences between or among groups was performed using analysis tools within GraphPad Prism 7 (GraphPad Software, Inc., La Jolla, CA). All data are shown as mean ± SD unless otherwise indicated. Statistical significance with p < 0.05 was considered significant with multiple comparison correction performed as indicated. Measurements were performed in triplicate unless otherwise indicated with at least 2 independent experiments.

## Results

### CCL20 RNA expression is increased in liver RNA from patients with NAFLD fibrosis

A genome-wide strategy was used to identify candidate genes whose expression was dysregulated in NAFLD fibrosis. Hepatic gene expression profiles were generated to identify genes differentially expressed between two histological NAFLD groups. Three pools of three liver RNA samples (nine total samples) from patients with extreme obesity who underwent intra-operative liver biopsy during bariatric surgery who had normal histology and three pools of three liver RNA samples (nine total samples) from patients whose liver histology was classified as grades 1–3 fibrosis (5/9 with bridging fibrosis, Additional file 1: Table S1) were analyzed using the human genome U133 microarray (Affymetrix). This pooling approach provides limited power [27], thus statistical analysis was not rigorously applied to the data but instead was used to select candidate genes for subsequent validation. Genes whose expression was the most up-regulated on the microarray with signal log ratios of > 3 (Table 1) included two immune related genes, C-C motif chemokine ligand 20 (CCL20) and defensin, alpha 1 (DEFA1), as well as a collagen, type XV, alpha 1 (COL15A1). CCL20 was the most up-regulated gene with a signal log ratio of 5.9.

**Table 1:** Transcripts showing the highest up-regulation measured using the U133 human genome array (Affymetrix) in severe fibrosis relative to normal histology

Gene	Gene name	SLR
CCL20	C-C motif chemokine ligand 20	5.9
STMN2	Stathmin 2	5.3
IGHM	Ig rearranged mu-chain gene V-N-D-N-J-region	4.1
RTN1	Reticulon 1	4.0
IGL	Immunoglobulin lambda gene locus DNA, clone:84E4	3.9
AKR1B10	Aldo–keto reductase family 1, member B10	3.8
LCK	Lymphocyte-specific protein tyrosine kinase	3.4
DEFA1	Defensin, alpha 1	3.4
COL15A1	Collagen, type XV, alpha 1	3.2

*SLR* signal log ratio

Based on these data, we then focused analysis on soluble immune-related genes using a PCR-based microarray assay to measure the expression of a panel of 86 human inflammatory cytokine and receptor genes using hepatic RNA samples from a separate set of patients (Additional file 1: Table S2) with severe fibrosis (N = 6) and normal histology (N = 6). CCL20 (Additional file 1: Table S3) was again the most up-regulated gene (23.5-fold). IL8 was the second most up-regulated gene, whose circulating levels have been reported to be increased in patients with extreme obesity and NAFLD fibrosis [28]. CCL20 was also identified as on of the top 20 most up-regulated genes by RNA-sequencing of 142 liver samples from another independent group of participants from the same cohort (manuscript under review).

**Fig. 1:**
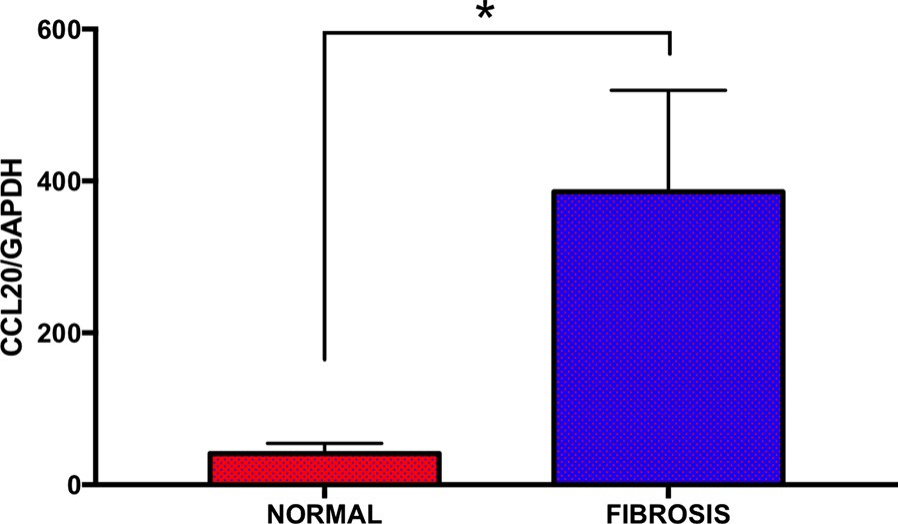
QPCR of CCL20 mRNA from 35 liver samples with normal histology (red bar) and 32 samples with fibrosis (blue bar) comprised of 11 with grade 1, 16 with grade 3 bridging fibrosis, and 5 with grade 4 cirrhosis. CCL20 mRNA levels were significantly increased (Mann–Whitney two-tailed p < 0.0001) in fibrosis

We then analyzed expression of CCL20 mRNA in an independent group of liver RNA samples with advanced fibrosis using quantitative PCR (QPCR). CCL20 mRNA expression from 32 samples with fibrosis (11 with grade 1, 16 with grade 3 bridging fibrosis, and 5 with grade 4 cirrhosis) was significantly increased (Mann–Whitney two-tailed p < 0.0001) relative to 35 liver samples with normal histology (Fig. 1).

### Circulating CCL20 protein levels are increased in patients with NAFLD fibrosis

**Fig. 2:**
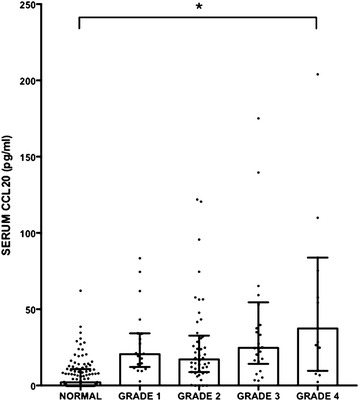
Box and whisker plot of serum CCL20 levels in 106 patients with normal liver histology (normal), 18 with grade 1 fibrosis (grade 1), 18 with grade 2 fibrosis (grade 2), 28 with grade 3 (bridging) fibrosis (grade 3), and 13 with grade 4 fibrosis/cirrhosis (grade 4). Three high CCL20 values, two grade 3 samples (270 and 538 pg/ml) and one grade 4 sample (580 pg/ml), are not shown in the plot for clarity of presentation. Median serum levels were increased in patient with histological grades of fibrosis (Kruskal–Wallis test Kruskal–Wallis one-way analysis-of-variance-by-ranks test p < 0.0001)

In order to determine whether liver RNA expression was associated with CCL20 protein expression in vivo, circulating CCL20 levels were determined. CCL20 protein levels in the serum were measured using ELISA in 183 patients (Additional file 1: Table S4) including 106 with normal liver histology, 18 with grade 1 fibrosis, 18 with grade 2 fibrosis, 28 with grade 3 (bridging) fibrosis, and 13 with grade 4 fibrosis (cirrhosis). As shown in Fig. 2, median levels were significantly increased in patients with fibrosis (one-way analysis-of-variance-by-ranks Kruskal–Wallis test p < 0.0001). The CCL20 level of each fibrosis group was also statistically higher (one-way analysis-of-variance-by-ranks Kruskal–Wallis test with Dunn’s adjusted p value p < 0.0001) than the normal group (normal 2.2 pg/ml, grade 1 fibrosis 19.3 pg/ml, grade 2 fibrosis 17.1 pg/ml, grade 3 fibrosis 24.7 pg/ml, grade 4 fibrosis 32.9 pg/ml).

**Fig. 3:**
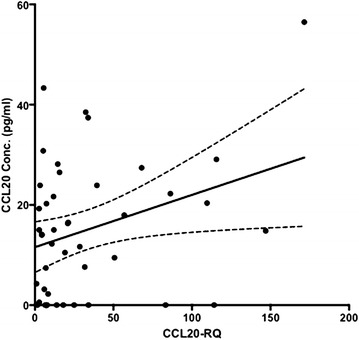
Plot of liver RNA expression of CCL20 versus CCL20 serum values. CCL20 liver RNA and serum levels were positively correlated. The 44 paired samples analyzed had an r^2^ of 0.1043 (r = 0.323) with a p (two-tailed) = 0.0325 and a regression line of y = 0.1043*x + 11.56

Data were available for 60 patients in whom both CCL20 liver RNA QPCR data and serum protein data were measured. Since correlations can be significantly influenced by outlier values, a robust regression and outlier removal procedure [29] was performed, which excluding outliers from either the liver RNA expression or CCL20 serum datasets. Correlation was then determined for 44 pairs of samples (Fig. 3). The r^2^ was 0.104 with a slope that was statistically different from zero (p = 0.032).

### CCL20 expression increases in response to fatty acid loading in LX-2 stellate cells

To investigate a potential cellular mechanism for the observed changes in vivo, we selected HepG2 cells as an in vitro model for human hepatocytes and LX-2 cells as an in vitro model for human hepatic stellate cells. Both cell lines have been well characterized in vitro systems for the study of NAFLD [30]. Cells were incubated with physiological levels [17] of palmitic acid (PA), oleic acid (OA), or a mixture of the two (PA/OA) for 24 h to induce lipid accumulation (Additional file 1: Figures S1 and S2). The highest increases in intracellular lipid staining in both HepG2 and LX-2 cells were observed in response to PA, followed by the PA/OA mixture then OA.

**Fig. 4:**
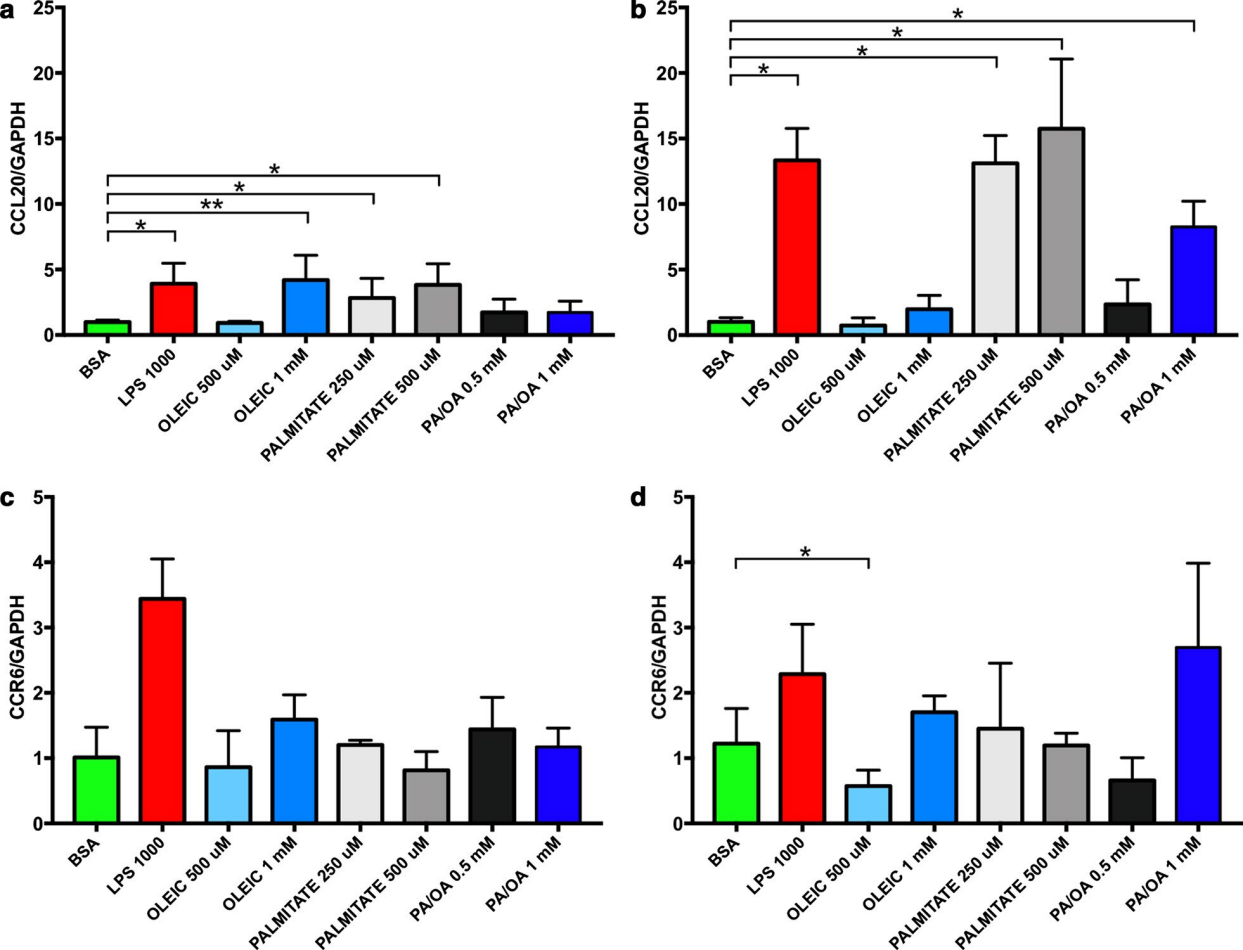
Transcript levels of CCL20 and CCR6 in HepG2 (**a**, **b**) and LX-2 (**c**, **d**) cells in response to fatty acid loading. **a** CCL20 transcript levels were increased by nearly fivefold (**Mann–Whitney p = 0.0029) in HepG2 cells treated with 1 mM oleic acid compared with BSA treated control cells, and by just over two- and threefold (*Mann–Whitney p < 0.0001), respectively, by 250 and 500 mM palmitic acid. **b** In contrast, in LX-2 cells, CCL20 levels increased ~ 13-fold with 250 μM (p < 0.0001) and the positive control LPS (p < 0.0001), ~ 15-fold with 500 μM of palmitic acid (p < 0.0001), and ~twofold (p < 0.0001) and ~eightfold (p < 0.0001) in response to oleic acid or 1 mM palmitic acid/oleic acid (50/50) mixture, respectively. **c** No statistically significant differences were observed in CCR6 transcript levels across the different treatments in HepG2 cells after adjusting for multiple comparisons. **d** In LX-2 cells, the only pairwise comparison to achieve statistical significance was ~ 50% decrease in CCR6 transcript levels in response to 500 μM oleic acid

Transcript levels of CCL20 and its cognate receptor CC chemokine receptor 6 (CCR6) were measured in lipid-loaded HepG2 and LX-2 cells using QPCR. Lipopolysaccharide (LPS), known to induce CCL20 expression in primary human hepatic stellate cells [31] was used as a positive control, which had no effect on lipid loading in either cell type (Additional file 1: Figures S1 and S2). In HepG2 cells (Fig. 4a), CCL20 transcript levels were found to be significantly increased (one-way analysis-of-variance-by-ranks Kruskal–Wallis test p < 0.0001) across treatments. In pair-wise comparisons, CCL20 transcript levels were significantly increased ~fivefold in response to 1 mM oleic acid (Mann–Whitney two-tailed test p = 0.0029), ~twofold in response to 250 μM palmitic acid (Mann–Whitney two-tailed test p < 0.0001), and ~threefold in response to 500 μM palmitic acid (Mann–Whitney two-tailed test p < 0.0001) and the positive control LPS (Mann–Whitney two-tailed test p < 0.0001). In contrast, in LX-2 cells (Fig. 4b), CCL20 levels increased ~ 13-fold with 250 μM (Mann–Whitney two-tailed test p < 0.0001) and the positive control LPS (Mann–Whitney two-tailed test p < 0.0001), ~ 15-fold with 500 μM of palmitic acid (Mann–Whitney two-tailed test p < 0.0001), and ~twofold (Mann–Whitney two-tailed test p < 0.0001) and ~eightfold (Mann–Whitney two-tailed test p < 0.0001) in response to oleic acid or 1 mM palmitic acid/oleic acid (50/50) mixture, respectively. No statistically significant differences were observed in CCR6 transcript levels across the different treatments in HepG2 cells (Fig. 4c) after adjusting for multiple comparisons. In LX-2 cells (Fig. 4d), the only pairwise comparison to achieve statistical significance was ~ 50% decrease in CCR6 transcript levels in response to 500 μM oleic acid (Mann–Whitney two-tailed test p = 0.041).

To determine if the increases in transcription of CCL20 resulted in increased CCL20 protein, ELISA was used to measure CCL20 in conditioned culture medium following 24 and 48 h of fatty acid or LPS treatment of LX-2 cells (Additional file 1: Figure S3). Approximately 50 pmol/ml and 22 pmol/nl of secreted CCL20 was observed in response to 500 μM palmitic acid and 250 μM palmitic acid, respectively, with almost 75 pmol/ml measured after LPS treatment. In response to treatment with a 50/50 palmitic acid/oleic acid mixture, a 1 mM concentration yielded approximately 16 pmol/ml protein, while 500 μM mixture produced < 2 pmol/ml. Treatment with oleic acid alone had no effect on CCL20 protein levels, similar to the results observed for the transcript measurements.

## Discussion

The initial hepatic steatosis that characterizes NAFLD results primarily from the storage of free fatty acids as triglycerides derived from plasma via the diet, de novo lipogenesis, and adipose tissue lipolysis, thus patients with extreme obesity are at particularly high risk [32]. Increased expression of the chemokine CCL20, which belongs to a class of molecules that serve as chemoattractants for the infiltration of immune cells to the injured liver [33], was associated with NAFLD fibrosis. CCL20 is a key molecule involved in the homing of dendritic cells, which have been shown to play a key role in liver fibrosis in animal models [34]. Our results extend initial observations of an approximate tenfold increase in expression of hepatic CCL20 transcript levels in eight samples from patients with NASH [31] to a large cohort of patients with extreme obesity. CCL20 also appears to play a key role in alcoholic liver disease [18], as well an emerging role in viral hepatitis [35, 36]. These data suggest that CCL20 may be a central regulatory molecule in a pathway common to the development of liver injury and fibrosis in alcoholic and non-alcoholic, as well as viral-mediated, liver disease.

CCL20, also known as macrophage inflammatory protein (MIP-3α) [37], serves as a ligand for its only receptor, CC chemokine receptor 6 (CCR6) [38]. In studies of alcoholic liver disease closely paralleling those reported here, CCL20 was found to be the most up-regulated chemokine gene by microarray gene expression profiling of human liver biopsy tissue [39]. This was followed by the findings that CCL20 hepatic RNA expression and serum levels were increased in patients with alcoholic hepatitis and were associated grade of hepatic fibrosis [31]. In that study, hepatic stellate cells were found to be the primary cell type producing CCL20, which was shown to induce a profibrogenic and proinflammatory expression profile in primary human hepatic stellate cells exposed to CCL20 in vitro. This implies a potential dual role for CCL20, one as a chemoattractant for immune cells, the other as an autocrine factor driving the fibrogenic process.

These data also point to HSCs as key cell type orchestrating liver damage. Induction of CCL20 in response to fatty acid loading suggests that HSCs may serve as a key lipid-sensing function in NAFLD, in addition to the presumed central role of the hepatocyte, in order to recruit dendritic and other immune cells as part of an inflammatory response to lipotoxicity. HSCs store triglycerides, as well as retinol, and express adipocyte-like features [40]. Further implicating stellate cells in lipid sensing is the association of a variant in the PNPLA3 gene, discovered through genome-wide association studies of NAFLD and associated with risk of NAFLD fibrosis [41], with increased lipid droplet content in primary HSCs and LX-2 cells, as well as with the increased production of several cytokines [42, 43]. Production of CCL20 by HSCs may therefore play a role in the mechanism of PNPLA3-mediated NAFLD.

An important aspect of our results, similar to those for the role of CCL20 in alcoholic liver disease, is the use of ex vivo human samples and cultured human cell lines to define the transcriptional regulation of the CCL20 gene in HSCs, which has not yet been well characterized in vivo [44]. Studies on humans are confounded by an uncontrolled environment, behavioral and genetic heterogeneity, and other variables. However, often used mouse models for either alcoholic or non-alcoholic liver disease lack physiological fidelity to humans. For example, available animal models do not re-capitulate the pathophysiology of severe alcoholic hepatitis that is found in humans [31]. Hepatic gene expression in nine NAFLD mouse models were found to have little overlap with transcriptional changes in human liver biopsies [45]. Even a dietary obese mouse model purporting a high level of fidelity to human NASH fibrosis was characterized by increased serum HDL levels and decreased triglyceride levels [46], the opposite of that found in the metabolic syndrome in humans [47], raising concerns about the potential contribution of these starkly different metabolic effects on physiological phenotype. Ambient temperature also appears to play a significant role in the development of NAFLD in mice [48]. Future studies on CCL20 using animal models of NAFLD may therefore be problematic due to these species-specific differences, thus other approaches, such as single cell RNA sequencing and multi-cellular in vitro models that based on human samples may be needed.

The results we obtained from a cohort of primarily females with extreme obesity who are seeking bariatric surgery may not be generalizable to individuals with lower levels of obesity where the pathophysiology of NAFLD may be different. However, the population with this degree of obesity constitutes over 6% of the US population and has been increasing [49]. The setting of bariatric surgery provides a unique opportunity to obtain liver biopsy tissue in the absence of suspected liver disease, which is largely the case for outpatient-based studies of liver tissue for which a clinical indication for biopsy must exist. Obtaining liver biopsies from individuals without suspicion of clinical disease, i.e., healthy subjects, is also more difficult due to the potential morbidity and mortality associated with the procedure. It is also possible that NAFLD in extreme obesity, which appears to be largely a clinically silent disease [32], may have a different pathophysiology than NAFLD in cohorts with lesser degrees of obesity. We also acknowledge that the relative homogeneity of the cohort, primarily of Caucasian ethnicity and from a single institution, and the retrospective design, may limit the applicability of the findings to other populations.

## Conclusion

CCL20 represents a new candidate molecule that may be involved in the pathogenesis of NAFLD fibrosis in the population with obesity that was investigated. Future studies on more heterogeneous populations will be needed in order to determine whether our findings are generalizable. As a soluble inflammatory mediator, CCL20 may also represent a potential diagnostic and/or therapeutic target.

## Additional file

**Additional file 1: Table S1.** Demographics and clinical characteristics of patients from whom samples were analyzed using Affymetrix Arrays. **Table S2.** Demographics and clinical characteristics of patients from whom samples were analyzed using the Human Inflammatory Cytokines and Receptors PCR Array. **Table S3.** Expression of genes in severe fibrosis relative to normal histology using the Human Inflammatory Cytokines and Receptors PCR Array. **Table S4.** Demographics and histological characteristics of patients from whom samples were analyzed using the CCL20 ELISA. **Figure S1.** Quantification of Oil Red O stained lipid-loaded HEPG2 cells. **Figure S2.** Quantification of Oil Red O stained lipid-loaded LX-2 cells. **Figure S3.** CCL20 protein levels in media of lipid-loaded LX-2 cells. **Figure S4.** CCL20 ELISA standard curve.

## Abbreviations

NAFLD: nonalcoholic fatty liver disease
CCL20: C-C motif chemokine ligand 20
CCR6: CC chemokine receptor 6
QPCR: quantitative PCR
