# Supplementary material for: CCL20 is up-regulated in non-alcoholic fatty liver disease fibrosis and is produced by hepatic stellate cells in response to fatty acid loading
Source: J Transl Med. 2018 Apr 24;16:108. doi: 10.1186/s12967-018-1490-y (PMC5937820; doi:10.1186/s12967-018-1490-y)
Supplement: Supplementary file 1 — Additional file 1: Table S1. Demographics and clinical characteristics of patients from whom samples were analyzed using Affymetrix Arrays. Table S2. Demographics and clinical characteristics of patients from whom samples were analyzed using the Human Inflammatory Cytokines and Receptors PCR Array. Table S3. Expression of genes in severe fibrosis relative to normal histology using the Human Inflammatory Cytokines and Receptors PCR Array. Table S4. Demographics and histological characteristics of patients from whom samples were analyzed using the CCL20 ELISA. Figure S1. Quantification of Oil Red O stained lipid-loaded HEPG2 cells. Figure S2. Quantification of Oil Red O stained lipid-loaded LX-2 cells. Figure S3. CCL20 protein levels in media of lipid-loaded LX-2 cells. Figure S4. CCL20 ELISA standard curve. [file 12967_2018_1490_MOESM1_ESM.docx]

**Additional file**

CCL20 is up-regulated in non-alcoholic fatty liver disease fibrosis and is produced by hepatic stellate cells in response to fatty acid loading.

*Chu, et al*

1. Table S1. Demographics and clinical characteristics of patients from whom samples were analyzed using Affymetrix Arrays.

2. Table S2. Demographics and clinical characteristics of patients from whom samples were analyzed using the Human Inflammatory Cytokines and Receptors PCR Array.

3. Table S3. Expression of genes in severe fibrosis relative to normal histology using the Human Inflammatory Cytokines and Receptors PCR Array.

4. Table S4. Demographics and histological characteristics of patients from whom samples were analyzed using the CCL20 ELISA.

5. Figure S1. Quantification of Oil Red O stained lipid-loaded HEPG2 cells.

6. Figure S2. Quantification of Oil Red O stained lipid-loaded LX-2 cells.

7. Figure S3. CCL20 protein levels in media of lipid-loaded LX-2 cells.

8. Figure S4. CCL20 ELISA standard curve.

**Table S1.** Demographics and clinical characteristics of Affymetrix Array study sample. Statistical significance was determined using a two-tailed, unpaired Student’s t test.

|  | **NORMAL**  **(N=9)** | **FIBROSIS (N=9)** | **P-value** |
| --- | --- | --- | --- |
| Female, n (%) | 7 (78) | 5 (56) | NS |
| Age at biopsy, years (median) | 46 | 56 | NS |
| BMI, kg/m2 (median) | 43.4 | 54.0 | 0.008 |
| Serum AST, U/L (median) | 21 | 43 | 0.005 |
| Serum ALT, U/L (median) | 22 | 77 | 0.003 |
| Glucose, mg/dL (median) | 90 | 114 | NS |
| HbA1c, % (median) | 6 | 7.4 | NS |
| Triglycerides, mg/dL (median) | 180 | 165 | NS |
| Total cholesterol, mg/dL (median) | 175 | 200 | NS |
| HDL-C, mg/dL (median) | 55 | 41 | 0.029 |
| Steatosis, grade (median) | 0 | 2 | <0.0001 |
| Lobular inflammation, grade (median) | 0 | 2 | <0.0001 |

**Table S2.** Demographics and clinical characteristics of the Human Inflammatory Cytokines and Receptors PCR Array study sample. Statistical significance was determined using a two-tailed, unpaired Student’s t test.

|  | **NORMAL**  **(N=6)** | **FIBROSIS (N=6)** | **P-value** |
| --- | --- | --- | --- |
| Female, n (%) | 6 (100) | 6 (100) | NS |
| Age at biopsy, years (median) | 46 | 48.5 | NS |
| BMI, kg/m2 (median) | 44.2 | 47.0 | NS |
| Serum AST, U/L (median) | 24.5 | 57.5 | 0.046 |
| Serum ALT, U/L (median) | 23 | 52 | 0.039 |
| Glucose, mg/dL (median) | 83 | 138.5 | 0.037 |
| HbA1c, % (median) | 5.6 | 9 | 0.011 |
| Triglycerides, mg/dL (median) | 109 | 383 | 0.015 |
| Total cholesterol, mg/dL (median) | 175 | 200 | NS |
| HDL-C, mg/dL (median) | 43.5 | 57 | 0.028 |
| Steatosis, grade (median) | 0 | 3 | 0.002 |
| Lobular inflammation, grade (median) | 0 | 2 | 0.002 |

**Table S3.**  Expression of genes in severe fibrosis relative to normal histology using the Human Inflammatory Cytokines and Receptors PCR Array. Statistical significance was determined using a two-tailed, unpaired Student’s t test.

| **Symbol** | **Description** | **Fold Change** | **p-value** |
| --- | --- | --- | --- |
| CCL20 | Chemokine (C-C motif) ligand 20 | 23.5 | 0.0015 |
| IL8 | Interleukin 8 | 9.2 | 0.001 |
| CCR4 | Chemokine (C-C motif) receptor 4 | 4.9 | 0.018 |
| IL13 | Interleukin 13 | 4.2 | 0.0414 |
| SPP1 | Secreted phosphoprotein 1 (osteopontin, bone sialoprotein I, early T-lymphocyte activation 1) | 4.1 | 0.0085 |
| LTA | Lymphotoxin alpha (TNF superfamily, member 1) | 4.1 | 0.0169 |
| CCL7 | Chemokine (C-C motif) ligand 7 | 4 | 0.0113 |
| CCL8 | Chemokine (C-C motif) ligand 8 | 3.8 | 0.0129 |
| IL1B | Interleukin 1, beta | 3.5 | 0 |
| LTB | Lymphotoxin beta (TNF superfamily, member 3) | 3.5 | 0.0175 |
| IL17C | Interleukin 17C | 3.4 | 0.0226 |
| CXCL14 | Chemokine (C-X-C motif) ligand 14 | 3.2 | 0.424 |
| CXCL3 | Chemokine (C-X-C motif) ligand 3 | 3 | 0.0908 |
| CCL18 | Chemokine (C-C motif) ligand 18 (pulmonary and activation-regulated) | 3 | 0.0935 |
| IL1F5 | Interleukin 1 family, member 5 (delta) | 2.9 | 0.0042 |
| TNF | Tumor necrosis factor (TNF superfamily, member 2) | 2.8 | 0.0011 |
| IL22 | Interleukin 22 | 2.8 | 0.0253 |
| CXCL10 | Chemokine (C-X-C motif) ligand 10 | 2.8 | 0.032 |
| IL1A | Interleukin 1, alpha | 2.7 | 0.0585 |
| IL1F7 | Interleukin 1 family, member 7 (zeta) | 2.5 | 0.0138 |
| CXCL9 | Chemokine (C-X-C motif) ligand 9 | 2.5 | 0.0258 |
| IL1F6 | Interleukin 1 family, member 6 (epsilon) | 2.5 | 0.0266 |
| CCL26 | Chemokine (C-C motif) ligand 26 | 2.5 | 0.0391 |
| CCL2 | Chemokine (C-C motif) ligand 2 | 2.5 | 0.0766 |
| CCR6 | Chemokine (C-C motif) receptor 6 | 2.5 | 0.114 |
| CCL19 | Chemokine (C-C motif) ligand 19 | 2.5 | 0.16 |
| CCR5 | Chemokine (C-C motif) receptor 5 | 2.4 | 0.0175 |
| IL9R | Interleukin 9 receptor | 2.4 | 0.0398 |
| CXCL5 | Chemokine (C-X-C motif) ligand 5 | 2.4 | 0.0452 |
| IFNA2 | Interferon, alpha 2 | 2.4 | 0.0709 |
| CCL21 | Chemokine (C-C motif) ligand 21 | 2.4 | 0.1354 |
| IL9 | Interleukin 9 | 2.3 | 0.0291 |
| CCR7 | Chemokine (C-C motif) receptor 7 | 2.3 | 0.1868 |
| CXCL11 | Chemokine (C-X-C motif) ligand 11 | 2.3 | 0.2146 |
| ICEBERG | ICEBERG caspase-1 inhibitor | 2.2 | 0.0324 |
| CCL17 | Chemokine (C-C motif) ligand 17 | 2.2 | 0.1419 |
| IL5RA | Interleukin 5 receptor, alpha | 2.1 | 0.2119 |
| BCL6 | B-cell CLL/lymphoma 6 (zinc finger protein 51) | 2 | 0.0098 |
| CXCL6 | Chemokine (C-X-C motif) ligand 6 (granulocyte chemotactic protein 2) | 2 | 0.0439 |
| IL1F10 | Interleukin 1 family, member 10 (theta) | 2 | 0.131 |
| CCL11 | Chemokine (C-C motif) ligand 11 | 2 | 0.2272 |
| IL8RB | Interleukin 8 receptor, beta | 2 | 0.2798 |
| CCR8 | Chemokine (C-C motif) receptor 8 | 2 | 0.2925 |
| IL8RA | Interleukin 8 receptor, alpha | 2 | 0.3385 |
| IL1F8 | Interleukin 1 family, member 8 (eta) | 1.9 | 0.1851 |
| CXCL13 | Chemokine (C-X-C motif) ligand 13 (B-cell chemoattractant) | 1.9 | 0.4067 |
| CCL1 | Chemokine (C-C motif) ligand 1 | 1.8 | 0.042 |
| CCR2 | Chemokine (C-C motif) receptor 2 | 1.8 | 0.1876 |
| CCL3 | Chemokine (C-C motif) ligand 3 | 1.7 | 0.1311 |
| SCYE1 | Small inducible cytokine subfamily E, member 1 (endothelial monocyte-activating) | 1.5 | 0.0312 |
| RTC | Reverse Transcription Control | 1.5 | 0.0766 |
| RTC | Reverse Transcription Control | 1.5 | 0.0816 |
| RTC | Reverse Transcription Control | 1.5 | 0.1025 |
| IL1F9 | Interleukin 1 family, member 9 | 1.5 | 0.3612 |
| CCL13 | Chemokine (C-C motif) ligand 13 | 1.5 | 0.4848 |
| IL10RB | Interleukin 10 receptor, beta | 1.4 | 0.0143 |
| PPC | Positive PCR Control | 1.4 | 0.1333 |
| CX3CR1 | Chemokine (C-X3-C motif) receptor 1 | 1.4 | 0.3678 |
| CCL5 | Chemokine (C-C motif) ligand 5 | 1.4 | 0.368 |
| CCR3 | Chemokine (C-C motif) receptor 3 | 1.4 | 0.4131 |
| PPC | Positive PCR Control | 1.3 | 0.2689 |
| CCL16 | Chemokine (C-C motif) ligand 16 | 1.3 | 0.36 |
| PPC | Positive PCR Control | 1.3 | 0.3916 |
| IL5 | Interleukin 5 (colony-stimulating factor, eosinophil) | 1.3 | 0.708 |
| C3 | Complement component 3 | 1.3 | 0.7825 |
| ABCF1 | ATP-binding cassette, sub-family F (GCN20), member 1 | 1.2 | 0.3401 |
| CXCL12 | Chemokine (C-X-C motif) ligand 12 (stromal cell-derived factor 1) | 1.2 | 0.4514 |
| CRP | C-reactive protein, pentraxin-related | 1.2 | 0.7005 |
| CD40LG | CD40 ligand (TNF superfamily, member 5, hyper-IgM syndrome) | 1.2 | 0.8583 |
| RPL13A | Ribosomal protein L13a | 1.1 | 0.2549 |
| ACTB | Actin, beta | 1.1 | 0.3122 |
| B2M | Beta-2-microglobulin | 1.1 | 0.4488 |
| IL13RA1 | Interleukin 13 receptor, alpha 1 | 1.1 | 0.4851 |
| IL10RA | Interleukin 10 receptor, alpha | 1.1 | 0.6257 |
| IL1R1 | Interleukin 1 receptor, type I | 1.1 | 0.6927 |
| IL1RN | Interleukin 1 receptor antagonist | 1.1 | 0.7575 |
| IL10 | Interleukin 10 | 1 | 0.9649 |
| GAPDH | Glyceraldehyde-3-phosphate dehydrogenase | -1 | 0.8589 |
| TOLLIP | Toll interacting protein | -1.1 | 0.4586 |
| XCR1 | Chemokine (C motif) receptor 1 | -1.1 | 0.7752 |
| C5 | Complement component 5 | -1.2 | 0.0947 |
| CCL15 | Chemokine (C-C motif) ligand 15 | -1.2 | 0.3699 |
| CCR1 | Chemokine (C-C motif) receptor 1 | -1.2 | 0.4724 |
| LTB4R | Leukotriene B4 receptor | -1.2 | 0.49 |
| MIF | Macrophage migration inhibitory factor (glycosylation-inhibiting factor) | -1.3 | 0.3636 |
| CXCL2 | Chemokine (C-X-C motif) ligand 2 | -1.3 | 0.6119 |
| HPRT1 | Hypoxanthine phosphoribosyltransferase 1 (Lesch-Nyhan syndrome) | -1.4 | 0.0951 |
| CXCL1 | Chemokine (C-X-C motif) ligand 1 (melanoma growth stimulating activity, alpha) | -1.4 | 0.33 |
| CCL4 | Chemokine (C-C motif) ligand 4 | -1.4 | 0.7933 |
| CCR9 | Chemokine (C-C motif) receptor 9 | -1.5 | 0.4087 |
| C4A | Complement component 4A (Rodgers blood group) | -1.6 | 0.0561 |
| CEBPB | CCAAT/enhancer binding protein (C/EBP), beta | -1.7 | 0.0376 |
| CCL24 | Chemokine (C-C motif) ligand 24 | -1.7 | 0.3634 |
| CCL23 | Chemokine (C-C motif) ligand 23 | -1.9 | 0.0187 |
| CCL25 | Chemokine (C-C motif) ligand 25 | -1.9 | 0.2679 |

**Table S4.** Demographics and histological characteristics of patients from whom samples were analyzed using the CCL20 ELISA.

|  | **NORMAL**  **(N=106)** | **GRADE 1**  **FIBROSIS (N=18)** | **GRADE 2 FIBROSIS (N=18)** | **GRADE 3**  **FIBROSIS**  **(N=28)** | **GRADE 4 FIBROSIS (N=13)** |
| --- | --- | --- | --- | --- | --- |
| Female, n (%) | 96 (91) | 15 (89) | 16 (89) | 17 (61) | 10 (77) |
| BMI, kg/m2 (mean) | 45.9 (7.6) | 48.7 (7.0) | 48.2 (10.1) | 49.1 (7.6) | 47.3 (9.8) |
| Steatosis, grade (median) | 0 | 2.7 (0.5) | 2.3 (0.7) | 2.4 (1.6) | 2 (1.2) |
| Lobular inflammation, grade (median) | 0 | 1.3 (0.5) | 1.7 (0.6) | 1.6 (0.7) | 1.2 (0.8) |

**Figure S1.** Oil Red O stained lipid-loaded LX-2 cells with quantification.


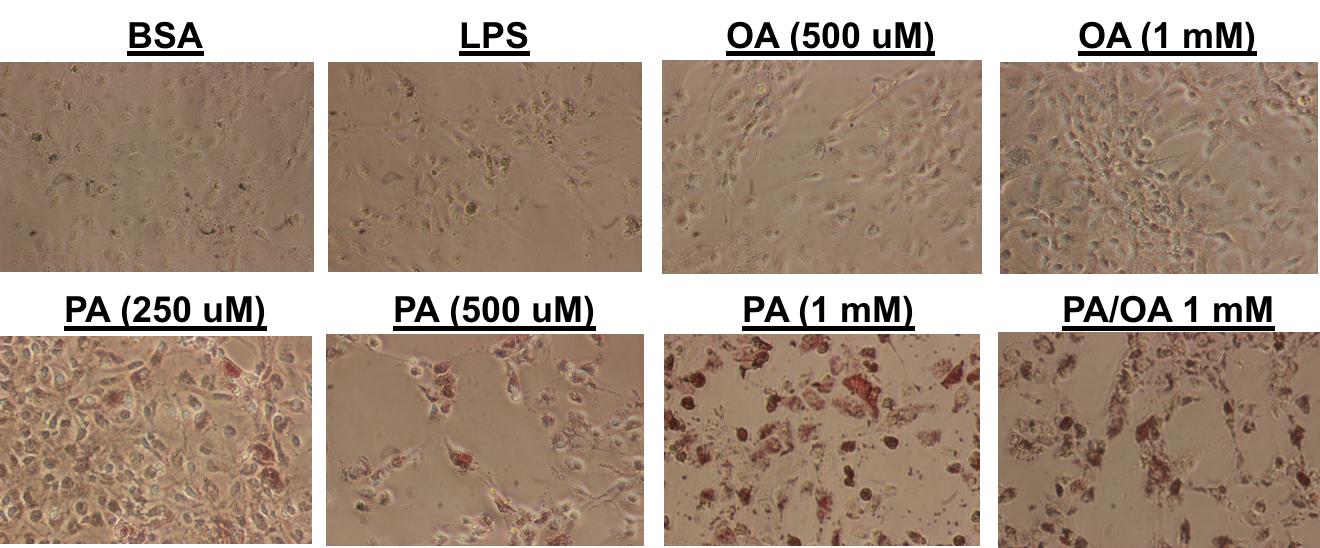


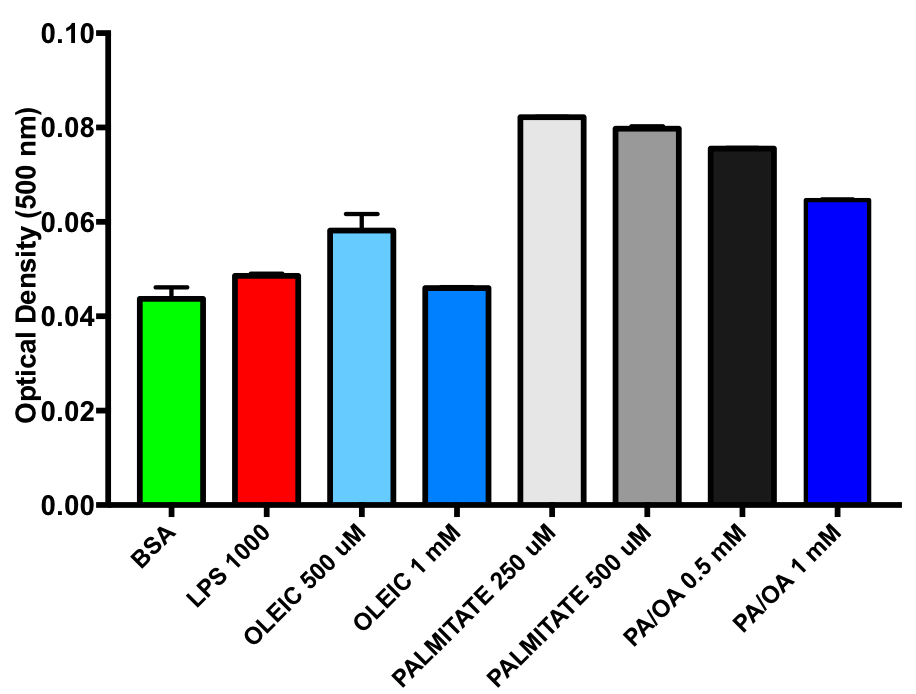


**Figure S2.** Quantification of Oil Red O stained lipid-loaded HEPG2 cells with example of HEPG2 staining.


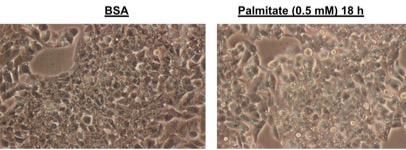


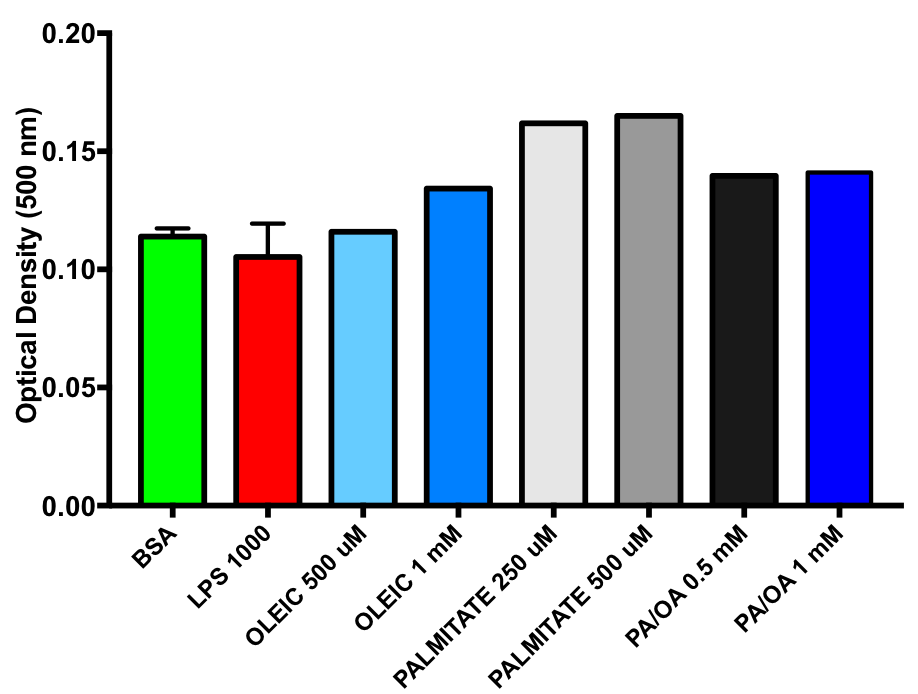


**Figure S3.** CCL20 protein levels in media of lipid-loaded LX-2 cells. CCL20 levels were highest in LPS and Palmitate treated cells, in parallel with the QPCR expression data in Figure 2. All measurements were performed in triplicate.


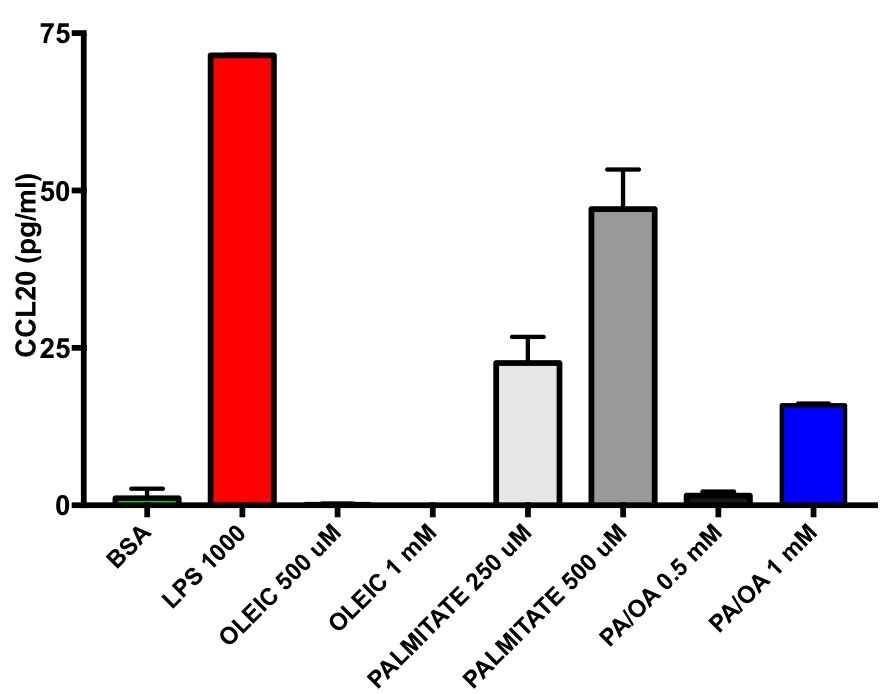


**Figure S4.** Standard Curve of CCL20 ELISA assay.
